# Triaging in Mass Casualty Incidents: A Simulation‐Based Scenario Training for Emergency Care Senior Residents

**DOI:** 10.1111/tct.70083

**Published:** 2025-03-25

**Authors:** Daisy Rotzoll, Claudia Pott, Robert Stöhr, Thomas Hartwig, André Gries

**Affiliations:** ^1^ Faculty of Medicine, Skills and Simulation Centre LernKlinik Leipzig University of Leipzig Leipzig Germany; ^2^ German Red Cross Local State Rescue School Leipzig Germany; ^3^ Protestant Deaconess Hospital Leipzig Germany; ^4^ Emergency Department University Hospital Leipzig Leipzig Germany

**Keywords:** emergency care, mass casualty incidents, scenario training, simulated patients, simulation, triaging

## Abstract

**Background:**

Mass casualty incidents (MCIs) are events where the number of patients may exceed the resources available for emergency management. In emergency departments (ED), MCIs go along with high patient allocation in a short time period. For ED staff, simulation‐based immersive scenario training can be an effective tool to develop communicative and leadership competencies to manage these situations.

**Approach:**

A novel simulation‐based immersion experience was developed for senior residents in emergency care. To our knowledge, this is the first scenario training focusing on in‐hospital triage and management of MCI in an ED using simulated patients, visual tracking, bodycams and active participant location tracking as educational tools. The participants were either directly involved by managing triage and allocation of in‐hospital resources for 14 patients or in remote observation of the running scenario on an audiovisual basis.

**Evaluation:**

After implementation of the pilot MCI simulation‐based scenario training, 13 participants completed surveys (48% response rate) including open‐ended response items. Quantitative data and open‐ended responses using an electronic response system were sequentially analysed to evaluate training feasibility and acceptability.

**Implications:**

We designed a novel simulation‐based MCI scenario training focusing on learning objectives involving confidence gain in ED triage. Although this format is extremely resource and time consuming, the highly positive evaluation (participants strongly agreed or agreed that the simulation scenario was suitable for in‐hospital MCI triage training) implies that this innovative education technique should be considered for future emergency medical services (EMS) training sessions.

## 1 Background

While disasters such as earthquakes or catastrophic floods are rare, out‐of‐hospital casualties such as train or bus accidents, poisonings or lightning strikes occur more frequently, leading to a sudden, simultaneous influx of high numbers of victims into regional emergency departments (EDs). To cope with such situations, numerous triage protocols have been developed to standardise initial prehospital assessment as well as in‐hospital reassessment with the purpose of maximising triage reliability in MCIs [1, 2]. The potential for incidents involving high numbers of patients needing treatment is omnipresent, leading to the necessity to train for such rare situations in different educational scenarios. Simulation plays an important role in the training of such scenarios for all healthcare professionals potentially involved in a MCI. While training of prehospital providers to master MCI triage skills has been studied extensively [3], data on ED scenario training focusing on management and team communication in MCI in EDs is scarce. Many emergency medical service (EMS) triage protocols do not give details as to how triage should be performed in resource‐constrained in‐hospital situations. Competencies in managing in‐hospital patient flow while treating large numbers of injured victims is essential for optimal patient care in such situations. Although simulation training programmes to support clinical judgement and decision‐making skills in an appropriate and timely manner during MCIs are available for prehospital triage [4], MCI simulations for optimising in‐hospital care focusing on communication and management skills in ED need to be further developed, especially for residents in charge of triage and management. We therefore developed a scenario training for medical doctors completing the German education programme for subspeciality licensing in clinical acute and emergency medicine [5], focusing on communication and management skills in an ED setting.

MCI simulations for optimising in‐hospital care focusing on communication and management skills in ED need to be further developed.

## 2 Approach

**FIGURE 1:**
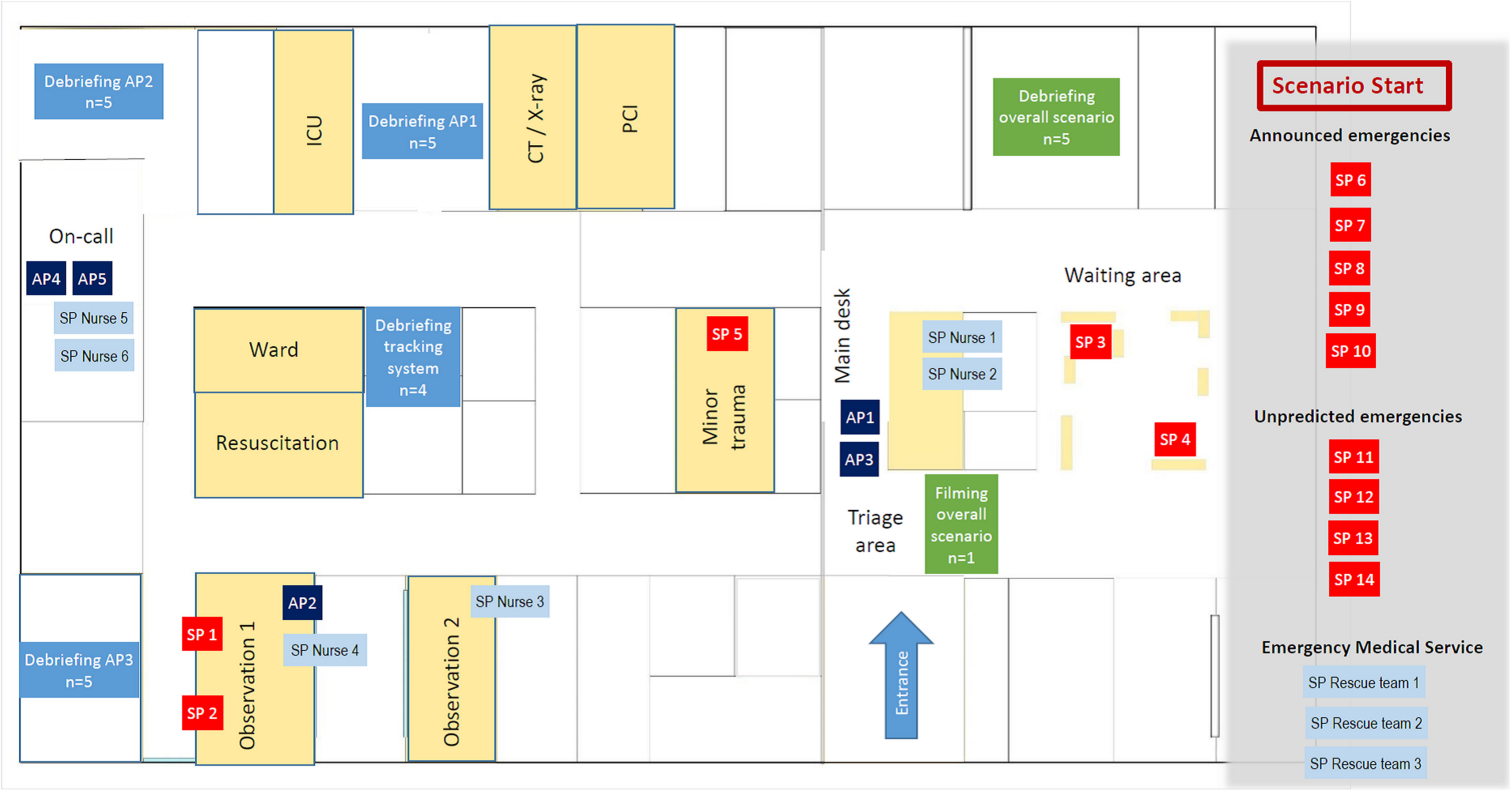
Room plan showing simulated ED and location of APs 1 to 5, SPs 1 to 5, SP Nurses 1 to 4 and location of debriefing groups at the start of the scenario. The number of announced emergencies SPs 6 to 10, of unpredicted emergencies SPs 11 to 14 and EMS SP rescue Teams 1 to 3 are marked to the left.

For the 6‐h training session (performed on 3 June 2022), a 30‐min MCI scenario involving a boat accident on a nearby lake was developed, leading to an unknown number of casualties. This MCI was chosen due to the fact that near to our simulation centre, many lakes have evolved from flooding former opencast coal mines, making this MCI situation all the more possible. The number of simulated patient (SP) casualties chosen (*n* = 14) encompasses a frequently seen amount and leaves many diagnoses open, such as trauma versus non‐trauma, intoxication and hypothermia. The location of the nearby lakes makes it likely that not too severely injured victims make their way on their own to the ER, leading to an influx of unpredicted emergencies (SPs 11 to 14). Figure 1 shows the set‐up of the simulated ED at the beginning of the scenario with SPs (SPs 1 to 5) and SP nurses involved (SP Nurse 1 to 6), as well as SPs (SPs 6 to 14) and SP rescue Teams 1 to 3 yet to be involved in the scenario. While a number of patients were already being treated or waiting to be treated in the simulated ED (SPs 1 to 5), the victims (SPs 6 to 14) were brought to the simulation centre ED by a SP rescue team (1 to 3) after being announced by the EMS‐dispatch centre. Further unpredicted emergencies (SPs 11 to 14) arrived without EMS assistance. The simulated emergency care unit comprised a triage area, a main desk required for coordination, a waiting area, a room for handling minor trauma patients, two patient‐observation rooms, a resuscitation room, a ward room and an intensive care unit (ICU), as well as a possibility to ask for x‐rays or a CT scan or percutaneous coronary intervention (PCI; see Figure 1). SP Nurses 1 to 4 were either in charge of administration (SP Nurse 1), triage (SP Nurse 2), patients in the emergency care unit (SP Nurses 3 and 4) or were on‐call (SP Nurses 5 and 6). Active participants (AP 1 to AP 5) were distributed according to the scenario script: AP 1 as emergency care head, AP 2/AP 3 as residents and AP 4/AP 5 as on‐call residents from ICU and surgery. Small‐group observation with four to five participants each were distributed in the simulation centre rooms for remote audiovisual observation of the scenario, accompanied by one facilitator per group. For clarification of the tasks to be performed by the active participants AP 1 to AP 5, the standard operating procedure (SOP) for MCI preparation was used, and for task clarification of the observers, a checklist and observer instructions were distributed (see Supporting information). The observation results were then discussed in the final plenary debriefing session led by the facilitators.

A total of 39 facilitators, SPs and technicians were involved in delivering the simulated scenario training (Table 1). The arrivals of announced and unpredicted emergencies SPs 6 to 14 were simulated in a way that redistribution of patients already being treated in the ED was unavoidable. For staff involved in the scenario training, a detailed role description was prepared to give minute‐by‐minute instructions. Materials such as SOPs relevant for emergency care and patient charts were created for each SP case 1 to 14 (see Supporting information for an exemplary role description, initial assessment protocol and lab work for SP 1 as well as the patient documentation sheet, SOP for MCI preparation as well as checklist and instructions for MCI observation; further material can also be obtained from the authors on request).

**TABLE 1:** Staff involved in the simulation‐based MCI triage scenario as facilitators, SPs, debriefing assistants and technicians (*n* = 39 in total).

Staff	*n*
Hospital staff (as facilitators or SP nurses)	7
Simulation centre lead (as facilitator)	1
Student tutors (as SPs or debriefing assistants)	11
German Red Cross local state rescue school (as facilitators, SPs and SP rescue teams)	14
Technicians	6

**FIGURE 2:**
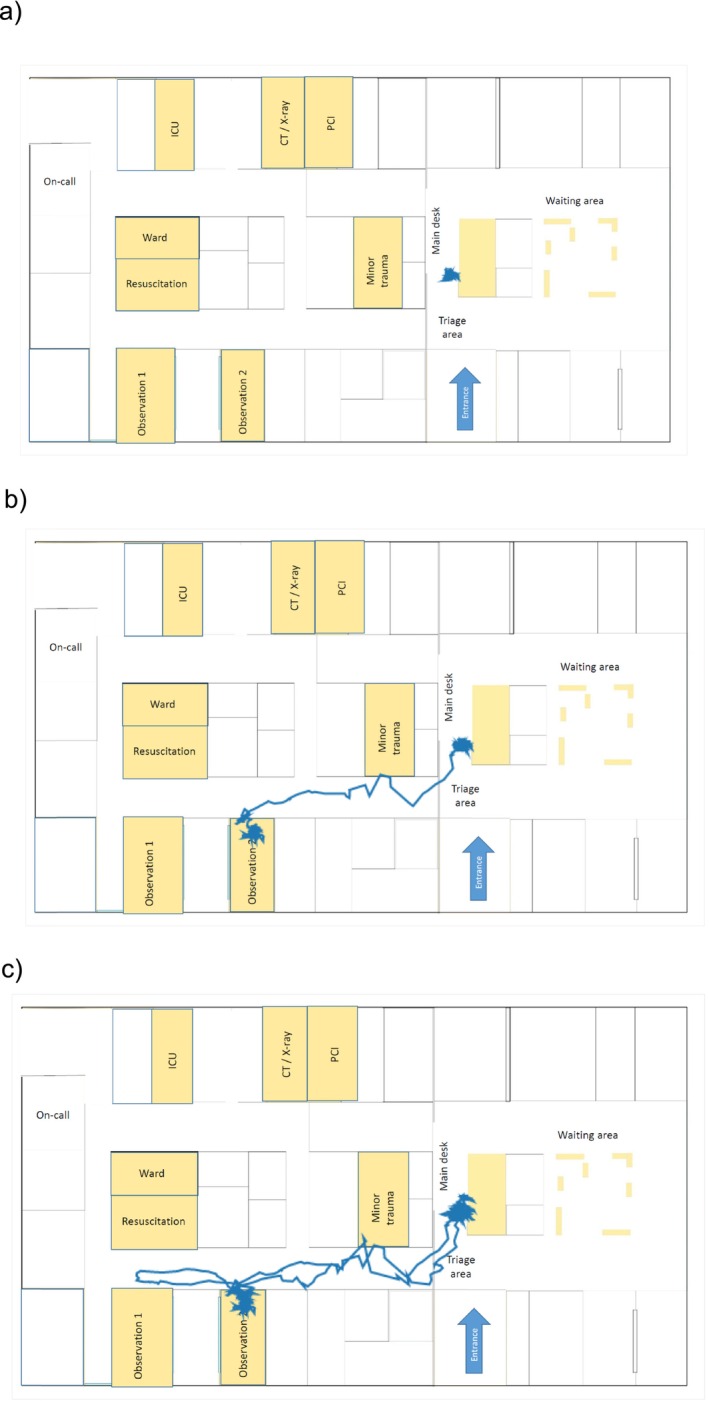
Example for the localisation system tracking analysis of AP 1 during the first 30‐min scenario training for time points Minute 1 (a), 15 (b) and 30 (c). During the scenario, AP 1 was mainly present in the triage area, while also being involved for a longer period of time in observation Room 2, and leaving the triage area in the midst of further patients arriving. Analysis of movement patterns was used for debriefing purposes after completion of the scenario.

**FIGURE 3:**
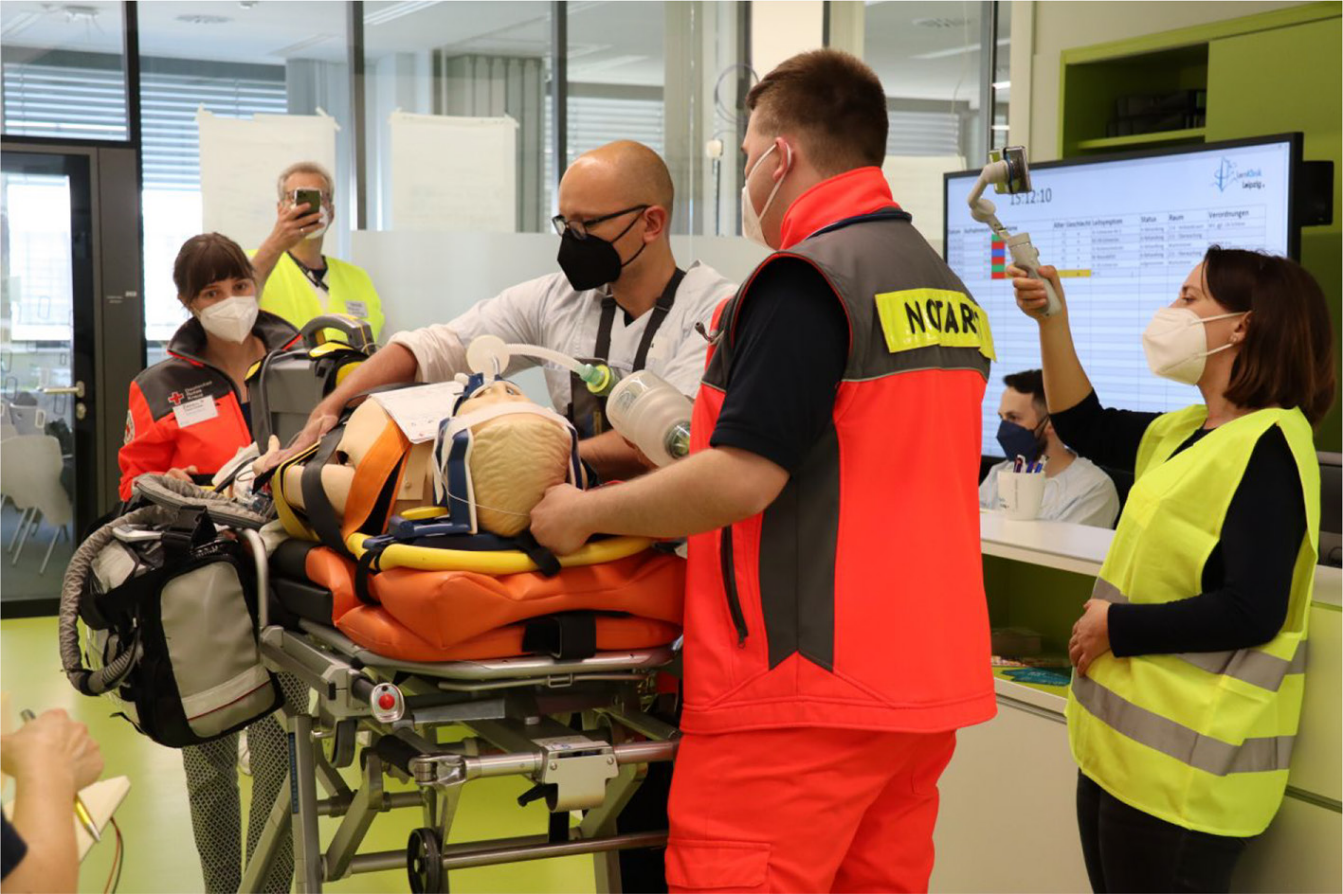
SP rescue Team 1 (wearing orange suits) handing over SP 1 (manikin) to participant AP 1 (third from left), with an observer participant (extreme right, yellow vest) filming the scene for later debriefing purposes. In the right back, see triage dashboard with triage levels listed.

**FIGURE 4:**
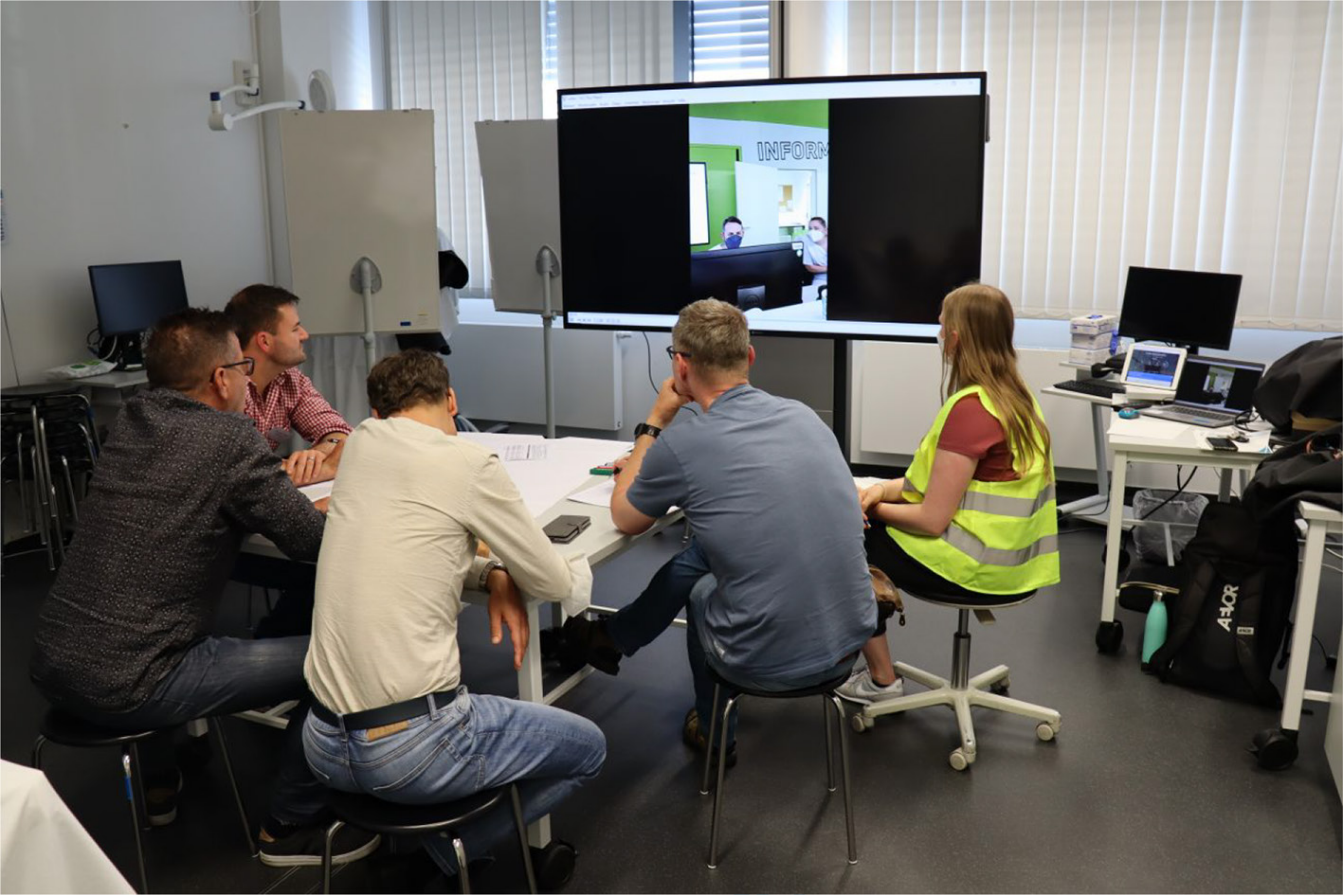
Participant observer group analysing the audiovisual camera tracking attached to the vest of AP 1, together with a student–tutor debriefing assistant (wearing yellow vest, extreme right).

Twenty‐seven participants were present, all being physicians in EDs at different hospitals ranging from university hospitals to small rural clinics. All were senior residents with leadership roles in their EDs or long‐standing clinicians in responsible positions. They were involved in a state training programme for acute and emergency care medicine with the described scenario training being part of the programme [5]. In the overall 6‐h simulation training (Table 2), the scenario was repeated twice with rotating roles for the active participants as well as the observing participants following the scenario via an audiovisual camera system in small groups in preparation of debriefing and feedback (Debriefing AP 1, AP 2, AP 3, tracking system and overall scenario, see Figure 1). Each small‐group debriefing was facilitated by a medical student trained as a debriefing assistant who had at least 2‐years' experience working as such in the simulation centre. Small‐group observation tasks included: observation of audiovisual recordings of portable cameras worn by AP 1, AP 2 and AP 3, observation of tracking data recorded by the overall tracking system for AP 1 to AP 5 as well as observation of an overall scenario film focusing on the action in the triage area, recorded by a transportable camera held by one of the participants (see Figures 1, 2 and 3). Great emphasis was put on the gathering of a structured group feedback for debriefing. Five debriefing groups focused either on giving AP 1 (see Figure 4), AP 2 or AP 3 structured feedback, or collecting important decision‐making data from the overall scenario film or from the tracking system.

**TABLE 2:** The MCI triage simulation scenario schedule (6 h total).

Schedule	Time line (in minutes)
Arrival and welcome	15
Scenario briefing	30
Role distribution among participants for Scenarios 1 and 2	10
Tour of simulation premises in small groups (*n* = 5–6 participants)	30
Distribution active participants and observer groups to starting points	10
Scenario 1	30
Preparation debriefing by observer groups	30
Debriefing (all participants and faculty)	60
Break and recreation	20
Distribution active participants and observer groups to starting points	5
Scenario 2	30
Preparation debriefing by observer groups	30
Debriefing (all participants and faculty)	60

## 3 Evaluation

An explanatory sequential mixed methods design was used to evaluate the feasibility and acceptability of our training session. For evaluation of the described simulation‐based MCI scenario training, a questionnaire with quantitative and qualitative data acquisition was developed using Evasys (Evasys, Lüneburg, Germany). The questionnaire consisted of questions used in the general quality management system of the medical faculty for course evaluation, modified and refined by the facilitator team, using a 5‐point Likert‐scale (*strongly agree* to *strongly disagree*), and open‐ended responses for feedback and comments on the training session. Thirteen out of 27 participants responded (48%).

### 3.1 Evaluation Results

**FIGURE 5:**
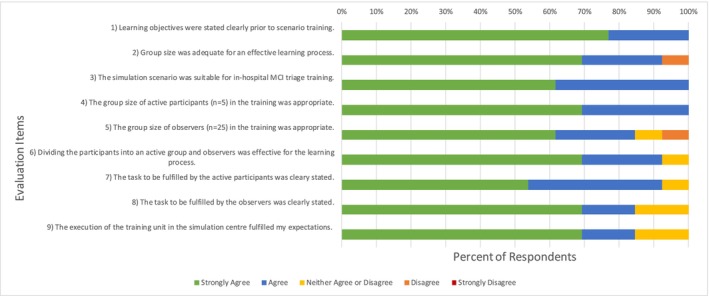
Evaluation of Items 1 to 9 focusing on simulation scenario feasibility and acceptability.

The questionnaire was distributed at the end of the 6‐h training session (see Table 1). Figure 5 shows nine essential recorded evaluation items. All participants strongly agreed or agreed that all learning objectives were stated clearly prior to the training, and 92% were of the opinion that the group size was adequate for an effective learning process. The scenario used for the ED‐triage was regarded as very suitable or suitable, as well as the group size of the active participants AP 1 to AP 5. Due to the big group of observers (*n* = 22), 7.7% found that this group size was not appropriate. Ninety‐two percent supported the participant division into a group of active participants and observers in charge of debriefing. The tasks to be fulfilled by active participants and observers were regarded as clearly stated by 92% and 85%, respectively. For 85%, the simulation scenario training fulfilled the purpose of training MCI management in the ED.

Quotes from the electronic questionnaires via open‐ended responses supported the quantitative data.

## 4 Implications

The importance of training for MCIs is well established and includes teaching relevant competencies such as organisation of in‐hospital treatment teams with the ability to re‐deploy team members efficiently [6]. Hospital surge capacity needs to be kept in mind where ad hoc measures have to be taken in hospitals. In Germany, hospitals are required by law to prepare, update and exercise for MCI situations [7]. In our scenario, the SOP for MCI preparation of the adjacent university hospital was adapted and used as preparation material for the participants of the MCI scenario training (see Supplementary Material ‘SOP for MCI preparation’). The implications of our simulation‐based immersion training session include demonstrating the overall usefulness of a simulation‐based scenario training for MCI triage. The simulation‐based immersion experience described here led to high satisfaction rates among the participants. Although most MCI educational trainings are recently based on either web‐based lectures, monograph reading exercises [8] or table‐top exercise templates [9, 10], making use of the growing material including VR modalities [6, 10, 11, 12], and face‐to‐face exercises [12, 13] are meanwhile rather the exception, making it all the more important to offer immersive scenario trainings for MCI preparedness as an experiential learning activity [14]. A unique feature in our scenario training was the implementation of an overall tracking system (see Figure 2), where each AP 1 to AP 5 was tracked during the 30‐min scenario. Figure 2c shows that AP 1 was only involved at the main desk as well as observation Rooms 1 and 2 throughout the entire scenario, while five announced and four unpredicted emergencies arrived in the meantime. The tracking data offered valuable discussion material for the final feedback as to how team responsibilities were defined or possibly not defined between APs 1 and 5 during the 30‐min scenario.

The simulation‐based immersion experience described here led to high satisfaction rates among the participants.

The learning activity proposed here comprised five AP roles in the scenario, while 22 participants focused on scenario observation and debriefing. In the plenary debriefing session, each observer group summarised three key moments on a flip chart to share, discuss and stimulate the final facilitator debriefing. Of the five categories to focus on in the observation (situational awareness, clinical decision making, leadership, communication and team, patient and employee safety: see Supporting Information ‘checklist for observers’), all observer groups focused on the categories ‘Communication and Team’ as well as ‘Leadership’ as being the most challenging; this was confirmed by the active participants. Although the overall satisfaction with the training programme was very high (Figure 5, evaluation Item 9), two participants each wished for adaptation of the group size with a shift to more active participants in the scenario training (Figure 5, evaluation Items 2 and 5). An adaptation of the scenario training with more role‐play participation of the residents (i.e., giving scenario‐play roles as SPs, SP nurses or members of the SP rescue teams to the participants) may therefore be an option to decrease staff as well as participant observation roles in the scenario, as has been done by Yu and Coffey [15]. A challenge for this training programme lies in the facilitator‐to‐participant ratio: 39 facilitators in total were involved for the MCI scenario training of 27 residents in total, giving a ratio of approximately 1.5 facilitators per participant. This ratio shows the immensely high commitment associated with this training, which should be taken into consideration when planning immersive scenarios for MCIs in the future. Further studies with assessment elements will be needed to document the long‐term effectiveness of such projects. Nonetheless, the high satisfaction of the participants and motivation of the facilitators should not be underestimated, and the diverse educational tools used to enable this MCI scenario training (SPs, technical simulators, video camera and bodycam implementation, tracking) can be a highly motivating factor for other centres to adopt this training in their institutions.

## Author Contributions

**Daisy Rotzoll:** conceptualization, investigation, writing – original draft, methodology, validation, visualization, writing – review and editing, formal analysis. **Claudia Pott:** project administration, data curation, supervision, resources. **Robert Stöhr:** project administration, data curation, supervision, resources. **Thomas Hartwig:** conceptualization, writing – review and editing, project administration, software, data curation, supervision, resources. **André Gries:** investigation, writing – review and editing, software, formal analysis, project administration, resources, supervision, data curation.

## Ethics Statement

This study was approved as exempt by the Research Ethics Board, University of Leipzig, Medical Faculty (correspondence to the first author from 1 February 2024).

## Conflicts of Interest

The authors declare no conflicts of interest.

## Supporting information


**Data S1** Supporting Information.

**Data S2** Supporting Information.

**Data S3** Supporting Information.

**Data S4** Supporting Information.

**Data S5** Supporting Information.

**Data S6** Supporting Information.

## Data Availability

The data that support the findings of this study are available from the corresponding author upon reasonable request.
