# Supplementary material for: Triaging in Mass Casualty Incidents: A Simulation‐Based Scenario Training for Emergency Care Senior Residents
Source: Clin Teach. 2025 Mar 25;22(3):e70083. doi: 10.1111/tct.70083 (PMC11937622; doi:10.1111/tct.70083)
Supplement: Supplementary file 1 — Data S1 Supporting Information. [file TCT-22-e70083-s003.pdf]

ERSTEINSCHÄTZUNGSPROTOKOLL

Zentrale Notfallaufnahme

MANV Szenario LernKlinik Leipzig

Universitätsklinikum Leipzig
Anstalt öffentlichen Rechts

**ZNAA1**<br>**Arndt**<br>Anton<br>**14.08.1986** M 35<br>Gottschallstraße 20<br>04157 Leipzig<br>**4658391 13783197**	**(Verdachts-) Diagnose**<br>OB-Schmerzen	**Vitalparameter bei Eintreffen**
		RRsyst/dia (mmHg): 150/90
	Symptombeginn ~ 6h	Herzfrequenz (/min): 110
	Transport: RTH (NAW) RTW KTW	O2 Sättigung (%): 97
	Übergabe an	Atemfrequenz (/min): 22
	Sr. NFA:	Blutentnahme: (JA) NEIN
	Arzt NFA:	12-Kanal-EKG: (JA) NEIN
Vor Weiterleitung an andere Behandlungseinrichtung obligate Rücksprache mit Arzt NFA!		Erfolgt: (JA) NEIN

**Schmerzen (VAS)**	10 9 (8) maximal, sehr stark	7 6 5 4 mäßig - stark	3 2 1 leicht	0 Kein Schmerz

ROT: Behandlung SOFORT

Atmung	Kreislauf	Bewusstsein	Blutung	weitere Symptome
fehlend	kein Puls	nicht ansprechbar	persistierend bzw. unstillbar	Gesichtsödem
akute und starke Atemnot	kaltschweißig	akute Vigilanzminderung	hoher Blutverlust	Pupillendifferenz
Stridor	zentralisiert			akute Sehstörung
Zyanose	HF < 40 /min	GCS 15		akute neurologische Ausfälle
O2 Sättigung < 90%	HF > 120/min			akuter Thoraxschmerz
	RR < 90 mmHg			(sehr starke Schmerzen (VAS 8-10))

GELB: Behandlung < 10 min.

Atmung	Kreislauf	Bewusstsein	Blutung	weitere Symptome
leichte-mäßige Atemnot	Puls arrhythmisch	sehr unruhig	Z.n. Bluterbrechen	Offene Fraktur
Fremdkörpergefühl	HF < 50 /min	akut desorientiert	Z.n. blutigem Stuhlabgang	Extremitäten Fehlstellung
path. Atemmechanik	HF >100 /min	deutliche Verhaltensauffälligkeit		Erbrechen
	RR>180 mmHg	Substanz- bzw. Drogeneinnahme		Reduzierte Sehschwäche
				Bericht über Bewusstlosigkeit
				Bericht über Kopfverletzung
				Chemikalienunfall
				Harnverhalt
				Hinweis auf Anämie
				mäßig-starke Schmerzen (VAS 4-7)

GRÜN - Behandlung < 60 min.

Atmung	Kreislauf	Bewusstsein	Blutung	weitere Symptome
normal	normal	vollständig	keine akute Blutung	deutliche Verschlechterung AZ
		orientiert		lokale Schwellung
				lokale Infektion Überwärmung
				leichte Schmerzen (VAS 1-3), akut

BLAU - Behandlung < 120 min.

Atmung	Kreislauf	Bewusstsein	Blutung	weitere Symptome
normal	normal	normal	keine akute Blutung	keine akuten Symptome

Fachbereich	Behandlungs - Priorität (Zutreffendes markieren)				Handzeichen
ZNA	(sofort)	< 10 min	< 60 min	< 120 min	
