# Supplementary material for: Triaging in Mass Casualty Incidents: A Simulation‐Based Scenario Training for Emergency Care Senior Residents
Source: Clin Teach. 2025 Mar 25;22(3):e70083. doi: 10.1111/tct.70083 (PMC11937622; doi:10.1111/tct.70083)
Supplement: Supplementary file 2 — Data S2 Supporting Information. [file TCT-22-e70083-s004.pdf]

[illegible]

Untersuchung	Ergebnis	Vorwerte	Referenzbereich Einheit	Material	*
**Hämatologie**					
**Kleines Blutbild**				EDTA-Vollblut	bk
Hämatokrit	0.437		0.4 - 0.53 l/l	EDTA-Vollblut	
Hämoglobin	9.10		8.4 - 10.9 mmol/l	EDTA-Vollblut	
Hämoglobin, g/dl	14.7		13.5 - 17.5 g/dl	EDTA-Vollblut	
Erythrozyten	4.92		4.5 - 5.9 exp 12/l	EDTA-Vollblut	
Verteilungsbreite Erythrozyten	11.8		< 15 %	EDTA-Vollblut	
mittl.korp.Erythrozytenvolumen	88.8		80 - 96 fl	EDTA-Vollblut	
mittl.korp.Hämoglobin d.Ery	1.85		1.7 - 2 fmol	EDTA-Vollblut	
mittl.korp.Hämoglobin d.Ery, pg	29.8		27.4 - 32.2 pg	EDTA-Vollblut	
mittl.korp.Hämoglobinkonzentration	20.80		20 - 22 mmol/l	EDTA-Vollblut	
mittl.korp.Hämoglobinkonzentr., g/dl	33.5		32.2 - 35.4 g/dl	EDTA-Vollblut	
Thrombozyten	308		140 - 360 exp 9 /l	EDTA-Vollblut	
Verteilungsbreite Thrombozyten	11.9		fL	EDTA-Vollblut	
mittleres Thrombozytenvol.	10.3		9.4 - 12.9 fl	EDTA-Vollblut	
Leukozyten	6.6		3.5 - 9.8 exp 9/l	EDTA-Vollblut	
**Elektrolyte**					
Natrium	141.7		135 - 145 mmol/l	Plasma	pc
Kalium	3.76		3.49 - 4.63 mmol/l	Plasma	
Kalzium, gesamt	2.47		2.15 - 2.50 mmol/l	Plasma	fc
**Herz/Muskel**					
Creatinkinase	2.37		0.63 - 2.91 µkat/l	Plasma	fc
**Leber/Pankreas**					
ALAT (GPT)	0.28		0.17 - 0.85 µkat/l	Plasma	fc
ASAT (GOT)	0.39		0.17 - 0.85 µkat/l	Plasma	fc
alkalische Phosphatase	0.93		0.67 - 2.15 µkat/l	Plasma	fc
Gamma-Glutamyltransferase	0.27		0.17 - 1.19 µkat/l	Plasma	fc
Bilirubin, gesamt	8.1		< 17.1 µmol/l	Plasma	fc
Lipase	↑13.13 ++		< 1 µkat/l	Plasma	fc
**Niere**					
Kreatinin (enzymatisch)	63.0		59 - 104 µmol/l	Plasma	
GFR nach CKD-EPI aus Kreatinin	123		> 90 ml/min/1.73m²	Plasma	

Abschätzung der glomerulären Filtrationsrate (GFR) mittels CKD-EPI- und MDRD-Formel:
- nur valide für Erwachsene (<70 Jahre)
- nicht bei akutem Nierenversagen anwendbar
- Übergewicht/erhöhte Muskelmasse sowie Untergewicht führen zur Unter- bzw. Überschätzung der GFR
- Korrekturfaktor für dunkelhäutige Patienten: x 1,159

Untersuchung	Ergebnis	Vorwerte	Referenzbereich Einheit	Material	*
CKD-EPI-Formel: nur anwendbar für einen GFR-Bereich 20-120 ml/min/1.73m² (Hinweise/Fragen unter Tel. 97-22221).					
Harnstoff	2.90		< 8.3 mmol/l	Plasma	fc
**Entzündung**					
C-reaktives Protein (CRP)	<0.60		< 5 mg/l	Plasma	it
**Anämiediagnostik / Blutbildung**					
Lactatdehydrogenase	3.51		2.25 - 3.75 µkat/l	Plasma	fc
**Schilddrüse**					
Thyreotropin (TSH)	0.732		0.4 - 3.77 mU/l	Plasma	ec
**Gerinnung**					
Quick-Wert (TPZ)	103		> 70 %	Citrat-Plasma	
INR	1.0			Citrat-Plasma	
aPTT	31.4		25 - 37 s	Citrat-Plasma	

* Methodenverfahren: bk: Sysmex XN-9000. ec: ECLIA,Roche. fc: Photometrie,Cobas. it: Immunturbodimetrie. pc: Potentiometrie,Cobas.
