# Supplementary material for: Triaging in Mass Casualty Incidents: A Simulation‐Based Scenario Training for Emergency Care Senior Residents
Source: Clin Teach. 2025 Mar 25;22(3):e70083. doi: 10.1111/tct.70083 (PMC11937622; doi:10.1111/tct.70083)
Supplement: Supplementary file 3 — Data S3 Supporting Information. [file TCT-22-e70083-s001.pdf]

Zentrale Notaufnahme

LernKlinik Leipzig

Name	Vorname
- /	
Geb.-Datum:	Fall-Nr.:
Alter bei Aufn.	Staats-angeh.:
Telefon Patient:	

**Allergien:** ja nein<br>Sonstige:	**Antikoagulanzien:** ja nein<br>Präparat::<br>**Diabetes:** ja nein<br>Präparat:	**Zuweisung :**<br>Selbst<br>Hausarzt<br>Klinik<br>RD<br>Notarzt<br>RTH<br>Hausnotfall
**Schwangerschaft:** ja nein unbekannt	**Stoma:** Stuhl Urin<br>sonst.:	
**Tetanusschutz:** ja nein unbekannt	**Isolation:** ja nein<br>Grund:	
**Hilfsmittel/ Implantate:** Kontaktlinsen Brille Schrittmacher Hörgerät Port TEP<br>Zahnprothese- oben - unten /		Pat. achtet auf Eigentum:

Leitsymptom:

ERSTBEURTEILUNG – Primary Survey

**A**	**AF:**		/min	
**B**	**SpO2:**		%	
**C**	**HF / RR**		min / mmHg	
**D**	**GCS**	**Augen öffnen**	**Verbale Antwort**	**Mot. Antwort**
	**Summe:**	4-spontan<br>3-Aufforderung<br>2-Schmerzreiz<br>1-keine	5-orientiert<br>4-verwirrt<br>3-inadäquat<br>2-unverständl<br>1-keine	6-Aufforderung<br>5-gezielt<br>4-ungezielt<br>3-Beugekrämpfe<br>2-Streckkrämpfe<br>1-keine
	**Pupillen**	RE: weit mittel eng<br>LI: weit mittel eng	LR RE: prompt verz. keine<br>LR LI: prompt verz. keine	
**E**	**Schmerz Temp**	VAS:	Temperatur:<br>Ort:	

Triage:

Triage:	0	10	30	90	120

Aufnahmedatum: Zeit:
Erster Arztkontakt: verantw. Arzt:

Symptomatik/ Anamnese:

Befund / Lokalbefund:

**Maßnahmen:**		**Bildgebende Diagnostik:**	**Labor-** verw. Barcode:		
Flexüle	SFK- Ch		Praxis 2	BZ	BG:
EKG	Monitoring		Trop T	Herz	EK:
O2-Sonde - l/min			weiteres:		

Sonstige Maßnahmen:

Messwerte:

Vitalfunktionen:

Zeit	RR	Puls	SaO_2	axil	rect.	Ohr	BZ	Medi./Infusion	Zeit	Applikation	Dosis
	mmHg	/min	l/min	°C	°C	°C	mnol/l				

Verlauf:

Therapie:

Diagnosen:

Weiteres Procedere / Therapieempfehlung

Patient entlassen nach Hause WB-Arzt:

Stationäre Aufnahme:

Unterschrift Arzt:

LernKlinik UKL – MANV Übung
-Notfallversorgung-
BSNR 94000000 / LANR 999999900
Liebigstrasse 23/25 , 04103 Leipzig
Telefon 0341 – 97-15164
