# Supplementary material for: Triaging in Mass Casualty Incidents: A Simulation‐Based Scenario Training for Emergency Care Senior Residents
Source: Clin Teach. 2025 Mar 25;22(3):e70083. doi: 10.1111/tct.70083 (PMC11937622; doi:10.1111/tct.70083)
Supplement: Supplementary file 4 — Data S4 Supporting Information. [file TCT-22-e70083-s002.docx]

# Kurzbeschreibung / Ziel

**SOP MANV ZNA (Übungsszenario)***

***(ausschließlich zur Verwendung i.R. des MANV-Moduls zum SLÄK-Kurs Klinische Akut- und Notfallmedizin)**

# Geltungsbereich/ Organisationseinheit

**Zentrale Notaufnahme**

# Durchführung

Diese SOP regelt das Vorgehen bei durch den Rettungsdienst angemeldeten Massenanfall von Notfallpatienten mit einem wahrscheinlichen Eintreffen von **> 4 zeitgleich durch den Rettungsdienst zugeführten Patienten („MANV ZNA“)**.

**Alarmierung**

Die Information über **„MANV ZNA“** erfolgt durch die Rettungsleitstelle per Fax beim diensthabenden Oberarzt der Zentralen Notaufnahme. Ggf. an anderer Stelle eingehende Anrufe müssen an den Oberarzt weitergeleitet werden.

**Vorgehen**

1. Aufnahme der durch die Rettungsleitestelle gegebenen Informationen (per Fax) und Dokumentation auf Tafel vor ADMIN-Bereich
2. Bei wahrscheinlichen MANV mit > 4 zeitgleich eintreffenden Patienten wird wie folgt vorgegangen:
3. Information **Krankenhauseinsatzleitung (Tel.: 17767)**
4. Information der im Hintergrund befindlichen Mitarbeiter*in (Pflege wird über die Bereichsärzte informiert)

- **Bereich Chirurgie Arzt: Tel 15225**
- **Bereich ITS Arzt: Tel 15198**

1. Informationen der Intensivstationen und des OP-Bereichs über **ITS/OP-Koordinators Tel: 17352**
2. **Räumung der Zentralen Notaufnahme (in Abstimmung mit ITS/OP-Koordinator (Bettenkapazitäten))**

- sofortige Verlegung der nicht kritischen Patienten auf die Stationen
- Aufnahmeverpflichtung von intensivpflichtigen Patienten auch ohne komplett vorliegende Befunde (Ausdrucke)
- Entlassung von potentiell ambulant verbleibenden Patienten
- Aufforderung der ggf. anwesenden Besucher das Krankenhaus bzw. die Zentrale Notaufnahme zu verlassen
- Sicherstellung der Behandlung der weiterhin in der Notaufnahme behandlungspflichtigen Patienten

1. Besetzung der in der ZNA kritischen Bereiche mit Vorbereitung auf MANV
2. Sicherstellung **Ersteinschätzung** bei Eintreffen
3. Administrative Erfassung und Sicherstellung **Dokumentation (Pat.-Liste ADMIN)**

# Mitgeltende Dokumente

keine

# Begriffe, Abkürzungen

keine
