# Supplementary material for: Triaging in Mass Casualty Incidents: A Simulation‐Based Scenario Training for Emergency Care Senior Residents
Source: Clin Teach. 2025 Mar 25;22(3):e70083. doi: 10.1111/tct.70083 (PMC11937622; doi:10.1111/tct.70083)
Supplement: Supplementary file 5 — Data S5 Supporting Information. [file TCT-22-e70083-s006.docx]

**Checklist for Performance AP 1 thru AP 5, MCI Triage Scenario**

| **Category** | **Factors** | **Observation** |
| --- | --- | --- |
| **Situational Awareness** | **Collecting information** |  |
|  | **Interpreting information** |  |
|  | **Predicting and considering the future** |  |
|  | **Identifying the priorities** |  |
| **Clinical Decision Making** | **Making the right decision among the options** |  |
|  | **Implementing the selected decisions at the right time** |  |
|  | **Using critical thinking skills** |  |
| **Leadership** | **Ability to assign tasks** |  |
| **Communication and Team** | **Communication skills among team members** |  |
|  | **Communication skills with patients** |  |
|  | **If available, communication skills with patients‘ relatives** |  |
|  | **Ability to work as a team** |  |
| **Patient and employee safety** | **Compliance with patient safety principles** |  |
|  | **Identity confirmation** |  |
|  | **Explaining the procedure and providing information** |  |
|  | **Taking standard precautions** |  |

Adapted from:

Uslu Y, Kocatepe V, Sagir O, Karabacak U. 2019. Hybrid Simulation in Triage Training. *International Journal of Caring Sciences* 12 (3):1626-1637.
