# Supplementary material for: Triaging in Mass Casualty Incidents: A Simulation‐Based Scenario Training for Emergency Care Senior Residents
Source: Clin Teach. 2025 Mar 25;22(3):e70083. doi: 10.1111/tct.70083 (PMC11937622; doi:10.1111/tct.70083)
Supplement: Supplementary file 6 — Data S6 Supporting Information. [file TCT-22-e70083-s005.pptx]

## Slide 1
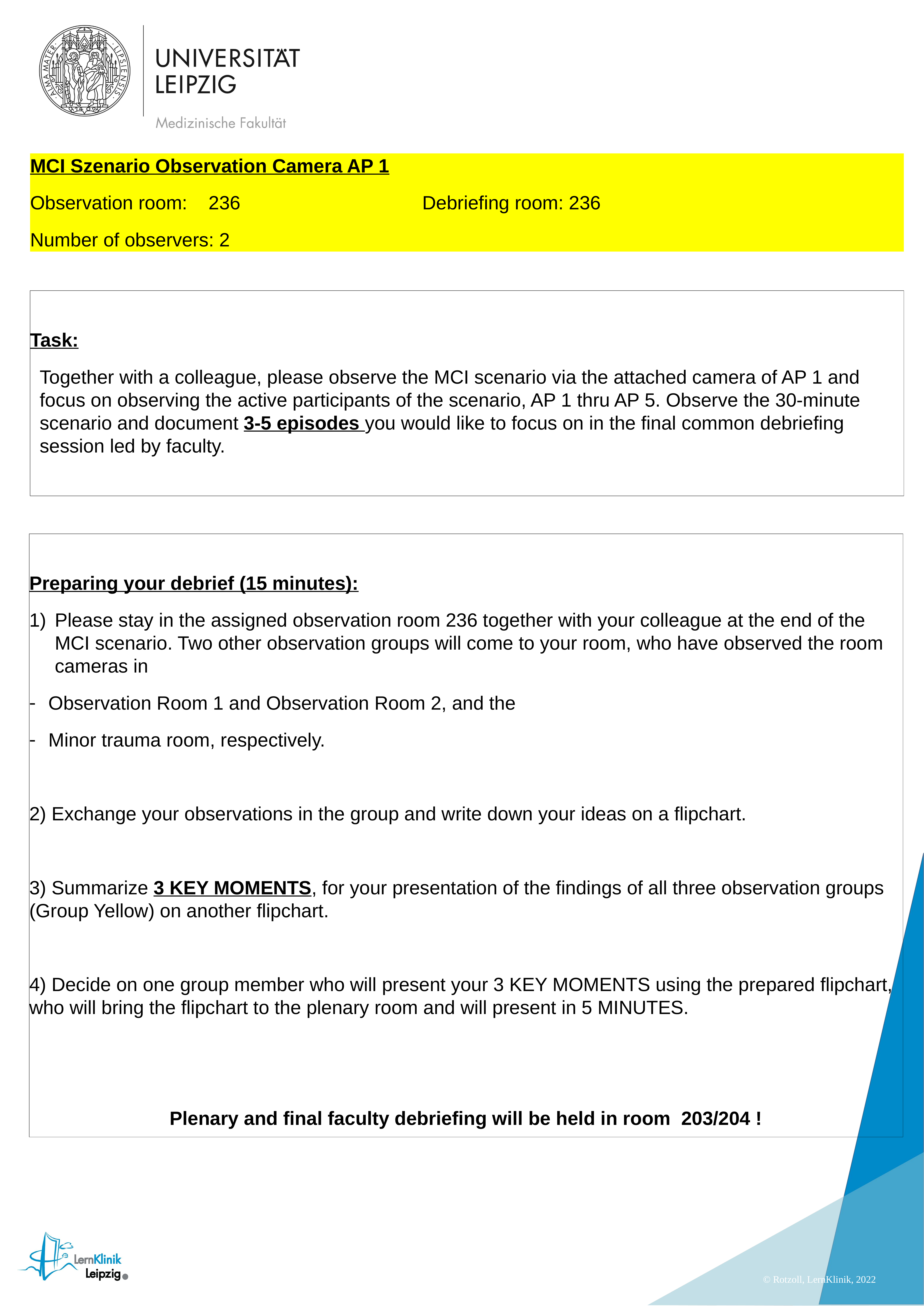

MCI Szenario Observation Camera AP 1
Observation room: 236								Debriefing room: 236
Number of observers: 2
Task:
	Together with a colleague, please observe the MCI scenario via the attached camera of AP 1 and focus on observing the active participants of the scenario, AP 1 thru AP 5. Observe the 30-minute scenario and document 3-5 episodes you would like to focus on in the final common debriefing session led by faculty.
Preparing your debrief (15 minutes):
Please stay in the assigned observation room 236 together with your colleague at the end of the MCI scenario. Two other observation groups will come to your room, who have observed the room cameras in
Observation Room 1 and Observation Room 2, and the
Minor trauma room, respectively.
2) Exchange your observations in the group and write down your ideas on a flipchart.
3) Summarize 3 KEY MOMENTS, for your presentation of the findings of all three observation groups (Group Yellow) on another flipchart.
4) Decide on one group member who will present your 3 KEY MOMENTS using the prepared flipchart, who will bring the flipchart to the plenary room and will present in 5 MINUTES.
Plenary and final faculty debriefing will be held in room 203/204 !
© Rotzoll, LernKlinik, 2022
